# Importance of social pharmacy education in Libyan pharmacy schools: perspectives from pharmacy practitioners

**DOI:** 10.3352/jeehp.2012.9.6

**Published:** 2012-03-14

**Authors:** Omar Saad Saleh Abrika, Mohammed Azmi Hassali, Abduelmula R Abduelkarem

**Affiliations:** 1 Discipline of Social and Administrative Pharmacy, School of Pharmaceutical Sciences, Universiti Sains Malaysia, Pulau Penang, Malaysia.; 2 Department of Clinical Pharmacy, College of Pharmacy and Health Sciences, Ajman University, Ajman, United Arab Emirates.

**Keywords:** Social pharmacy, Education, Curriculum, Libya

## Abstract

The present study aims to explore the perceptions among pharmacy practitioners in Libya on the importance of social pharmacy education. A qualitative methodology was employed to conduct this study. Using a purposive sampling technique, a total of ten Libyan registered pharmacists were interviewed. Based on the content analysis of the interviews, two major themes emerged, namely the understanding of social pharmacy education and the need for incorporating social pharmacy courses into the pharmacy education curriculum. The majority of the respondents knew about the concept. Of those that had no prior knowledge of this term, half of them expressed interest in knowing more about it. There was a positive perception of introducing social pharmacy into the undergraduate curricula among the respondents, and they believed that it is necessary for future pharmacists to know about social pharmacy components. The findings from the pharmacy practitioners' evaluation suggest the need to incorporate social pharmacy courses into the curricula of all pharmacy schools in Libya.

Social pharmacy has been concisely defined as a discipline concerned with the behavioral sciences relevant to the utilization of medicine by both consumers and healthcare professionals [1]. In extension to the behavioral and psychological aspects related to pharmacy, an area's pharmacy administration such as pharmacy management and marketing were also understood as fundamental components in social pharmacy [2]. The World Health Organization (WHO), through a consulting group pinpointed seven roles to which future pharmacists should aspire, namely caregivers, decision-makers, communicators, leaders, managers, life-long learners, and teachers [3]. Within this context, there is a need for future pharmacists to be trained in all aspects related to social pharmacy as it provides background for being involved in patient-oriented services.

A few developing countries, such as Malaysia have also recognized that pharmacies have the potential to provide enhanced contributions to primary health care, and have considered social pharmacy to be an important subject [3, 4]. Since the 1992-93 academic year, social pharmacy related courses have been included into the undergraduate pharmacy curriculum the Universiti Sains Malaysia [5]. In Malaysia, the introduction of social pharmacy courses into the study of pharmacy has been recognized for successfully increasing students' knowledge of human behavior, and the development of models for studying patient and consumer behavior.

In Libya, students selected based on the grades earned in secondary school (after 11 years total of primary and secondary schooling) need to undergo a one-year pre-pharmacy course at the Faculty of Science in a university. The faculty offers a four-year program leading towards a bachelor's degree in pharmacy [6]. The four-year program including the pre-pharmacy course encompasses the teaching of professional subjects and also includes three sessions of an 8-10 week summer training in the fields of community pharmacy, hospital pharmacy, clinical pharmacy, and pharmaceutical technology conducted at the end of the first, second, and third years of the four-year program.

The present study aims to document whether there is a need for social pharmacy courses during the undergraduate period in Libya based on interviews of pharmacy practitioners.

Using a semi-structured interview guide, interviews were conducted with a purposive sample of practitioners for 30-45 minutes per participant between June and September 2010. The interviews were conducted in the English language. Recruitment of practitioners continued until the point of saturation was reached, whereby no new additional themes emerged from participants in the last three interviews [7, 8]. Face-to-face interviews were conducted at a place and time that was convenient for the participants. Content analysis was used to identify the themes, and the transcripts were coded accordingly. The findings presented related to the understanding of social pharmacy education and the need for incorporating social pharmacy courses into pharmacy education in Libya.

Appropriate probing questions were used when necessary as has been done elsewhere [9]. To draw out more complete ideas from the participants, at the end of the interview session they were given the freedom to express additional views on the subjects discussed. Thematic content analysis was used to analyze the interview data with the goal of accurately identifying patterns or regularities within the data [10].

As part of the ethical requirements for this study, before the commencement of the interviews, we obtained the written informed consent of the participants. The demographic characteristics of the ten participants of the study were as follows: two females and eight males; four hospital pharmacists, two community pharmacists, and two drug store pharmacists; ages ranging from 31 to 50 years old.

The thematic content analysis of the interviews identified two major themes: the understanding of social pharmacy education and the need for incorporating social pharmacy courses into pharmacy education. There was one sub-theme: the suggestion of implementing social pharmacy subjects.

## The understanding of social pharmacy education

The interview guide questions were focused on the participants' understanding of social pharmacy education (see Appendix 1). A total of ten practitioners were asked whether they understood the concept of social. Six of the respondents reported that they had either heard of or had an idea about the concept of social pharmacy. Four claimed that they had never heard of the concept of social pharmacy. However, only two out of four expressed their willingness to learn about this concept. The new emerging subjects within social pharmacy such as social and administrative pharmacy have been widely offered to pharmacy undergraduates in developed countries. However, while it appeared that the majority of the respondents relied on the internet to obtain information about social pharmacy, it was more difficult to identify the specific types and sources of information used by the pharmacists.

The pharmacists were asked to rate their responses to the question, "What components of social pharmacy can you name." Four of the respondents had no idea about it and regretted not being able to answer the question. However, the other six respondents were able to describe certain topics that are commonly taught in social pharmacy courses such as communication and counseling, pharmacoeconomics, pharmacoepidemiology, pharmaceutical policy, and pharmacy ethics.

## The need for incorporating social pharmacy courses in pharmacy education

The respondents were asked about the idea of teaching components of social pharmacy to undergraduate pharmacy students in Libya. Four reported that this was the first time that they had heard about social pharmacy, and eight of the respondents agreed with the statement that it would be a good idea to teach components of social pharmacy to undergraduate pharmacy students in Libya. One of the respondents reported having a very limited idea about the concept of social pharmacy and wanting to know more about the concept. Some of the respondents fit in more than one of these subgroups.

The respondents were asked to identify the level of the curriculum at which various social science disciplines and subjects including psychology, anthropology, and economics were being taught to undergraduates in their alma mater. Only one reported that some of the basic knowledge of psychology was offered to the undergraduate pharmacy students during their preliminary year. However, the other nine practitioners reported that their pharmacy programs did not cover any of these topics during any level of study.

In response to the question about the importance of social pharmacy for improving communication with patients, eight respondents agreed that it was important. However, two did not agree that social pharmacy is important to improve communication with their patients.

## Suggestion for the implementation of social pharmacy subjects

To investigate the strategies to adopt for the future implementation of social pharmacy education in Libya, the respondents were asked about the necessity of offering social pharmacy education to the next generations of pharmacists. The majority of the respondents' answers were diverse but they can be grouped into two types. Eight suggested that social pharmacy or some of its components should be added to the current pharmacy curriculum as elective undergraduate courses, whereas two judged it necessary to consider these courses as compulsory. Three thought that such an implementation would require qualified local or foreign staff. The remaining two argued for the need to first understand the current pharmacy education strategies in developed countries and adapt them for local use.

In the present study the idea of teaching of social pharmacy courses to current pharmacy students seemed to be appreciated by the current pharmacy practitioners. The majority of the pharmacists interviewed fully supported the introduction of social pharmacy subjects in the undergraduate pharmacy curricula. Most of the respondents agreed that an education in social pharmacy has the potential to improve knowledge, skills, practice, and thus professionalism.

These reasons indicated that the Libyan pharmacy practitioners we interviewed are in line with arguments that have been expressed in previous studies on the necessity of teaching social pharmacy. The present study also found that components of pure social science subjects such as psychology, anthropology, and economics were not taught very much, if at all, or were poorly represented in undergraduate pharmacy curricula in Libya. Social pharmacy regularly draws upon the disciplines of sociology, social psychology, psychology, political science, educational studies, communications, economics, history, and anthropology. It leans more heavily on psychology, social psychology, sociology, political science, and economics, especially as these relate to issues in public health and social politics [11].

Despite the fact that two of the pharmacists interviewed did not agree that social pharmacy is important to improve communications with their patients, the other eight agreed. Furthermore, as with the introduction of any new course there will some personal and contextual barriers for implementation, adoption, and acceptability [5, 12]. In a nutshell, the findings of this qualitative research have provided insight into the need for social pharmacy courses in Libyan pharmacy curricula. The use of qualitative research for uncovering issues concerning the need for introducing social pharmacy courses also allowed for obtaining richer information from the participants despite the small sample size [13].

The main limitation of this study is basically inherent to the use of the qualitative methodology. Qualitative studies have always been subject to a lack of generalizability. This study was based on a limited sample of qualitative face-to-face interviews with Libyan pharmacists from two faculties, but the use of the qualitative methodology was the best approach for exploring the issue of interest in depth prior to engaging in large quantitative studies.

In conclusion, the present study showed that a group of Libyan pharmacy practitioners had positive views regarding the incorporation social pharmacy subjects in pharmacy education because it will enhance the their present professional and leadership roles. Therefore, pharmacy educators can use the findings in this study to actively consider incorporating social pharmacy subjects into the existing pharmacy curriculum.

## References

[1] Wertheimer A (1991). Social/behavioural pharmacyd: the Minnesota experience. J Clin Pharm Ther.

[2] Manasse HR, Rucker TD (1984). Pharmacy administration and its relationship to education, research and practice. J Soc Adm Pharm.

[3] (1997). The role of of the pharmacist in the health care system. Preparing the future pharmacist: curricular development [Internet]. World Health Organisation.

[4] Yeole PG, Puranik MP (2006). Pharmacy in India: at crossroads. Health Adm.

[5] Ibrahim MI, Awang R, Razak AD (1998). Introducing social pharmacy courses to pharmacy students in Malaysia. Med Teach.

[6] Javaid KA, Qazi TU, El-Migirab S (1981). Pharmaceutical education in Libya. Am J Pharm Educ.

[7] Morse J, Field A (1995). Qualitative research methods for health professionals.

[8] Glazer BG, Strauss AL (1997). The discovery of grounded theory: strategies for qualitative research.

[9] Berg B (2004). A dramaturgical look at interviewing: qualitative research methods for social sciences.

[10] Sandelowski M (2000). Whatever happened to qualitative description?. Res Nurs Health.

[11] Sorensen CA, Wood B, Prince EW (2003). Race and ethnicity data: developing a common language for public health surveillance in Hawaii. Californian J Health Promot.

[12] Ab Rahman AF, Ibrahim MI, Bahari MB, Nik Mohamd MH, Awang R (2002). Design and evaluation of the pharmacoinformatics course at a pharmacy school in Malaysia. Drug Inf J.

[13] Smith F (2002). Qualitative interviews: research methods in pharmacy practice.

